# Determining the longitudinal validity and meaningful differences in HRQL of the PedsQL™ Sickle Cell Disease Module

**DOI:** 10.1186/s12955-017-0700-2

**Published:** 2017-06-12

**Authors:** Julie A. Panepinto, J. Paul Scott, Oluwakemi Badaki-Makun, Deepika S. Darbari, Corrie E. Chumpitazi, Gladstone E. Airewele, Angela M. Ellison, Kim Smith-Whitley, Prashant Mahajan, Sharada A. Sarnaik, T Charles Casper, Larry J. Cook, Julie Leonard, Monica L. Hulbert, Elizabeth C. Powell, Robert I. Liem, Robert Hickey, Lakshmanan Krishnamurti, Cheryl A. Hillery, David C. Brousseau, for the Pediatric Emergency Care Applied Research Network (PECARN)

**Affiliations:** 1 Medical College of Wisconsin, Pediatric Hematology and Oncology, and the Children’s Hospital of Wisconsin, 8701 Watertown Plank Road, MFRC Suite 3050, Milwaukee, WI 53226 USA; 2 Johns Hopkins University, Pediatric Emergency Medicine, Baltimore, MD USA; 3 Children’s National Medical Center, Pediatric Hematology and Oncology, Washington, DC USA; 4 Baylor College of Medicine/Texas Children’s Hospital, Pediatric Emergency Medicine, Houston, TX USA; 5 Baylor College of Medicine/Texas Children’s Hospital, Pediatric Hematology and Oncology, Houston, TX USA; 6 Children’s Hospital of Philadelphia, Pediatric Emergency Medicine, Philadelphia, PA USA; 7 Children’s Hospital of Philadelphia, Pediatric Hematology and Oncology, Philadelphia, PA USA; 8 Wayne State University/Children’s Hospital of Michigan, Pediatric Emergency Medicine, Detroit, MI USA; 9 Wayne State University/Children’s Hospital of Michigan, Pediatric Hematology and Oncology, Detroit, MI USA; 10 University of Utah/Pediatric Emergency Care Applied Research Network Data Coordinating Center, Salt Lake City, UT USA; 11 Nationwide Children’s Hospital, Pediatric Emergency Medicine, Columbus, OH USA; 12 Washington University School of Medicine, Division of Pediatric Hematology and Oncology, St. Louis, MO USA; 13 Ann & Robert H. Lurie Children’s Hospital of Chicago, Division of Emergency Medicine, Northwestern University Feinberg School of Medicine, Chicago, IL USA; 14 Ann & Robert H. Lurie Children’s Hospital of Chicago, Hematology, Oncology & Stem Cell Transplant, Chicago, IL USA; 15 Children’s Hospital of Pittsburgh of University of Pittsburgh Medical Center, Pediatric Emergency Medicine, Pittsburgh, PA USA; 16 Department of Pediatrics, Aflac Cancer and Blood Disorders Center, Emory University School of Medicine, Atlanta, GA USA; 17 Department of Pediatrics, Division of Hematology/Oncology, University of Pittsburgh School of Medicine, Pittsburgh, PA USA; 18 Medical College of Wisconsin, Pediatric Emergency Medicine, and the Children’s Hospital of Wisconsin, Milwaukee, WI USA

**Keywords:** Sickle cell disease, Quality of life, Acute pain crises, Longitudinal validity, Responsiveness

## Abstract

**Background:**

Detecting change in health status over time and ascertaining meaningful changes are critical elements when using health-related quality of life (HRQL) instruments to measure patient-centered outcomes. The PedsQL™ Sickle Cell Disease module, a disease specific HRQL instrument, has previously been shown to be valid and reliable. Our objectives were to determine the longitudinal validity of the PedsQL™ Sickle Cell Disease module and the change in HRQL that is meaningful to patients.

**Methods:**

An ancillary study was conducted utilizing a multi-center prospective trial design. Children ages 4–21 years with sickle cell disease admitted to the hospital for an acute painful vaso-oclusive crisis were eligible. Children completed HRQL assessments at three time points (in the Emergency Department, one week post-discharge, and at return to baseline (One to three months post-discharge). The primary outcome was change in HRQL score. Both distribution (effect size, standard error of measurement (SEM)) and anchor (global change assessment) based methods were used to determine the longitudinal validity and meaningful change in HRQL. Changes in HRQL meaningful to patients were identified by anchoring the change scores to the patient’s perception of global improvement in pain.

**Results:**

Moderate effect sizes (0.20–0.80) were determined for all domains except the Communication I and Cognitive Fatigue domains. The value of 1 SEM varied from 3.8–14.6 across all domains. Over 50% of patients improved by at least 1 SEM in Total HRQL score. A HRQL change score of 7–10 in the pain domains represented minimal perceived improvement in HRQL and a HRQL change score of 18 or greater represented moderate to large improvement.

**Conclusions:**

The PedsQL™ Sickle Cell Disease Module is responsive to changes in HRQL in patients experiencing acute painful vaso-occlusive crises. The study data establish longitudinal validity and meaningful change parameters for the PedsQL™ Sickle Cell Disease Module.

**Trial Registration:**

ClinicalTrials.gov (study identifier: NCT01197417). Date of registration: 08/30/2010

**Electronic supplementary material:**

The online version of this article (doi:10.1186/s12955-017-0700-2) contains supplementary material, which is available to authorized users.

## Background

With the use of valid and reliable measures of health-related quality of life (HRQL), it has been well established that many patients with sickle cell disease have significant impairment in HRQL when in their baseline state of health [1]. The ability of a HRQL instrument to detect true change in health status over time (longitudinal validity) and knowledge of what change is meaningful to patients are both critical elements when using HRQL instruments to measure this patient-centered outcome. Recently the PedsQL™ Sickle Cell Disease module has been shown to be a feasible, reliable, and valid disease specific measure of HRQL [2]. However, the ability of this instrument to detect change in patients with sickle cell disease and knowing whether that change in HRQL is meaningful have not been determined. Understanding whether a change in HRQL reflects a meaningful change in health status for a patient is critical to support use of the measure in clinical trials and in the clinical care of patients.

The objectives of this paper are to determine the longitudinal validity of the PedsQL™ Sickle Cell Disease module and the change in HRQL that is meaningful. We hypothesized that the module would be responsive and would detect improvement in HRQL that was meaningful in patients who were experiencing an acute vaso-occlusive painful episode, specifically in the relevant domain of pain. Our secondary objectives were to determine the responsiveness of the PedsQL™ Generic Core Scales and Multidimensional Fatigue Module in these patients. We hypothesized that these instruments would detect change in the physical functioning and general fatigue domains.

## Methods

### Study setting and subjects

This study was conducted as an ancillary study within a multi-center randomized, double-blind, placebo controlled trial from December, 2010 to March, 2014 [3]. Eight clinical centers in the United States that are part of the Pediatric Emergency Care Applied Research Network (PECARN) participated in the study of patients ages 4–21 years who had sickle cell disease, specifically hemoglobin SS disease or hemoglobin Sβ^0^ thalassemia and who presented to the emergency department with a painful vaso-occlusive crisis. Patients were eligible for this HRQL study if they were admitted to the hospital for management of a painful vaso-occlusive crisis, enrolled in the parent trial, and completed HRQL measures for at least two of the HRQL measurement time points [3, 4]. For this ancillary HRQL study, all patients were combined into a single cohort of youth ages 4–21 years with hemoglobin SS or hemoglobin Sβ^0^ thalassemia to allow for examination of HRQL change over time because there were no differences between placebo and treatment groups in the primary (clinical endpoints) and secondary outcomes (HRQL) of the clinical trial. Details describing HRQL between placebo and treatment groups in the clinical trial are published elsewhere [3, 4].

The institutional review boards of each clinical center approved the study and consent for participation was obtained from all subjects and their parents. The study was registered at ClinicalTrials.gov (study identifier: NCT01197417).

### Measurements

Demographic and medical information was obtained on all patients through parent report and review of the child’s medical record.

The *Peds QL™ Sickle Cell Disease Module* is a 43 item module that encompasses nine scales including pain [2]. The module uses a 5-point Likert response scale (0 = never a problem to 4 = almost always a problem) that is reverse scored and transformed to a 0–100 scale (0 = 100, 1 = 75, etc.) so that a higher score indicates better HRQL. The Total Score and the scale scores are calculated by summing the items and dividing by the number of items answered. Per the developer’s instructions for scoring, at least 50% of the items in a scale must be answered to compute a scale score. The module has parallel reports for parent proxy (age ≥ 2 years) and child self report (age ≥ 5 years). For children ages 5–7 years the module was completed with the help of the research coordinator using a 3 point Likert scale as recommended by the PedsQL™ Administration Guidelines.

The *PedsQL™ Generic Core Scale* is a 23 item module that encompasses 4 scales. Its response scale and scoring are similar to the PedsQL™ Sickle Cell Disease module. The generic module also has two summary scores, Physical Health Summary (same as Physical Functioning Scale) and Psychosocial Health Summary (sum of items answered in the emotional, social, and school functioning scales divided by total number of items answered) [5]. The *PedsQL™ Multidimensional Fatigue Scale* is an 18 item measure that includes three scales that measure different aspects of fatigue. The scales are scored similarly to the Peds QL™ Sickle Cell Disease Module scales [5].

### Procedure

The HRQL measures were completed in paper form or by report over the phone by both the parent and child at three time points in this study 1) in the Emergency Department after the decision to admit the patient was made, 2) by phone one week post hospital discharge and 3) One to three months post discharge at a follow-up clinic visit.

To determine the patient’s perception of their improvement, at the one week post hospital discharge assessment, children were also asked to report how much they perceived their pain had improved in the week following hospital discharge via a global assessment of change question.

### Analyses

Demographic and clinical characteristics of the cohort were calculated using descriptive statistics. Longitudinal validity was calculated using two distribution-based methods and an anchor-based method. Distribution based methods rely solely on statistics and do not account for the patient’s perception of clinical change. An anchor based method takes into account an expected meaningful change that is then anchored to the change in HRQL score. Lastly, to determine statistical significance of change in HRQL over time, a linear mixed-effects model was utilized. This multi-pronged approach for longitudinal validity has been recommended in the literature [6]. Analyses were performed using SAS Version 9.3 (SAS Institute, Cary, NC).

### Distribution-based methods to support responsiveness

For the distribution-based methods, the effect size and the standard error of measurement (SEM) were calculated. The effect size was calculated to determine the magnitude of the group differences for the two follow-up time points as follows: the mean change in HRQL scores between 1) one week post discharge from the hospital and Emergency Department divided by the standard deviation of the scores from the Emergency Department and 2) One to three months post hospital discharge and Emergency Department divided by the standard deviation of the scores from the Emergency Department. Using Cohen’s statistics, an effect size of 0.2 is small, 0.5 is moderate, and >0.8 is large [7].

The SEM was used to determine what an important change in score is for an individual and was calculated as follows: SEM = σ $\sqrt{1-\alpha}$

where σ is the standard deviation of the baseline HRQL score and α is the reliability (Cronbach’s alpha) of the measure. The SEM represents the within-person variability over time. One SEM is an estimate of a significant change that has been shown repeatedly in prior research to correspond to an anchor-based change threshold for an individual, supporting responsiveness, in HRQL [8, 9].

### Anchor-based methods to support meaningful change in HRQL

#### Global assessment of change

For the anchor-based assessment, an anchor to pain was used at one week post discharge to anchor whether the change in HRQL was meaningful to patients. Specifically, patient’s completed the following global assessment of change in pain question: “Since you left the hospital, how has your pain been?” Patients self reported their response as: “No pain since discharge”, “Much better”, “A little better”, “The same”, “A little worse”, Much worse”. Parents provided proxy reported responses to the same global assessment of change in pain question. This anchor was chosen as it is relevant to patients, measures their improvement in pain globally, and is interpretable [10].

Mean change in HRQL scores for the Total scores for all three PedsQL™ measures and for the scores from the pain scales of the disease specific measure were determined for the following collapsed categories of global assessment of change in pain: “No pain since discharge”, “Much better”, “A little better”, and “The same or worse”. The categories of “The same, A little worse, and Much worse” were combined as worse pain was expected to be a rare occurence in this study setting. An anchor specific to the remaining PedsQL™ HRQL scales (for example the Emotional scale) were not assessed as part of this study.

A mean change in HRQL score that fell into the category of “A little better” was considered the minimum change that was meaningful. A mean change in HRQL score that fell into the categories of “Much better” and “No pain since discharge” were considered moderate to large meaningful changes in HRQL.

A Spearman rank correlation coefficient was calculated to assess the relationship between the clinical anchor (global assessment of change question) and the target HRQL domain.

#### Multivariable model to analyze HRQL over time

A linear mixed-effects multivariable model was used to analyze the impact of age, hydroxyurea use, study site, gender, and disease severity on HRQL (child self-report total HRQL) over time (Emergency Department and 1-week post-discharge). HRQL at 1–3 months was considered the baseline HRQL and included as a covariate. A random intercept was included for subject, as well as a random term for change in score between Emergency Department and one-week follow-up. Age, measured in years, was selected as a covariate because older children experience more frequent acute sickle cell pain crises and have longer length of hospital stay. Patient-reported hydroxyurea within three months of admission was included to examine the potential it may have to moderate HRQL during an acute sickle cell pain crisis. Because severity of sickle cell disease, study site, and gender could also affect HRQL, these variables were also included in the model. Children were classified as having severe disease if they had a history of acute chest syndrome and/or 3 or more hospitalizations for acute sickle cell pain events in the prior 3 years consistent with criteria we have used in the past [2, 11, 12]. All others were classified as having mild disease. Total scores from three measures of HRQL were each considered as an outcome in separate models: the PedsQL™ Sickle Cell Disease, the PedsQL™ Multidimensional Fatigue Scale score, and the PedsQL™ Generic Core Scale.

#### Cumulative distribution curves

In accordance with what has been recommended by others [13, 14] and to make the data more meaningful to clinicians, cumulative distribution function graphs were used to illustrate change in HRQL from Emergency Department presentation to one week follow-up for the total HRQL scores for each measure and for each of the three pain scales on the Sickle Cell Disease Module (Pain Impact, Pain and Hurt, Pain Management scales). Cumulative distribution curves present the full spectrum of change for the study population and allow for varied change thresholds or responder thresholds to be determined from these graphs [13].

## Results

A total of 187 patients (Table 1) were enrolled in the study. Ninety-four percent of patients were black and 4% were Latino. Eighty-nine percent had either been hospitalized 3 or more times in the last 3 years and/or had a history of acute chest syndrome constituting a population with more severe disease. Details on the proportion of patients with missing HRQL are provided in the Additional file 1: Tables S1, S2 and S3. Overall, HRQL scores were able to be calculated for over 80% of the patients for each time point. Approximately 20% of patients had missing global change in pain data.

**Table 1:** Baseline characteristics

Patients	Overall Group (*n* = 187)
Disease type	
Hgb SS	174 (93%)
Hgb Sβ^o^	13 (7%)
Age, mean (sd)	13.5 (4.5)
Age group	
4–11 years	74 (40%)
12–21 years	113 (60%)
Sex	
Female	94 (50%)
Treated with hydroxyurea within 3 months prior to randomization	114 (61%)
History of acute chest syndrome	143 (76%)
Hospitalizations for a pain crisis in past 3 years	
0	16 (9%)
1	23 (12%)
2	25 (13%)
3	29 (16%)
4	17 (9%)
5	13 (7%)
≥ 6	63 (34%)

### Distribution based methods

#### Effect sizes support responsiveness to change (longitudinal validity)

At the one week post discharge and one to three months post discharge times points, the PedsQL™ Sickle Cell Disease module was responsive to change (Table 2) in all scales but the Worry II and Communication I scale. The effect size was 0.74 and 0.70 for the Total Score at the two follow up time points respectively supporting moderate responsiveness to change.

**Table 2:** Child self report health-related quality of life: effect sizes

	ED Visit		1 Week			1–3 Months		
Domain	N	Mean (SD)	N	Mean	Effect Size	N	Mean	Effect Size
Sickle cell disease module								
Total Score	166	48.3 (15.87)	139	59.4	**0.74**	153	59.9	**0.70**
Pain and Hurt	165	49.8 (18.89)	137	66.6	**0.91**	152	62.7	**0.66**
Pain Impact	165	33.3 (19.53)	139	48.3	**0.84**	151	49.8	**0.78**
Pain Management and Control	163	41.8 (28.24)	137	54.3	**0.45**	151	52.3	**0.37**
Worry I	165	48.7 (23.75)	137	56.1	**0.34**	151	62.5	**0.54**
Worry II	143	69.4 (28.17)	120	74.8	**0.23**	130	75.2	0.19
Emotions	164	47.5 (34.51)	138	58.6	**0.39**	152	61.0	**0.37**
Treatment	163	55.7 (20.54)	137	60.6	**0.27**	151	61.0	**0.27**
Communication I	162	70.9 (27.55)	136	74.2	0.13	149	73.1	0.08
Communication II	158	45.0 (28.96)	133	56.3	**0.42**	144	59.1	**0.52**
Fatigue module								
Total Score	157	53.8 (18.53)	134	61.0	**0.41**	144	62.1	**0.47**
General Fatigue	158	52.0 (22.20)	135	60.0	**0.39**	145	64.0	**0.54**
Sleep/Rest Fatigue	157	48.6 (20.65)	134	57.1	**0.46**	144	59.2	**0.54**
Cognitive Fatigue	155	60.3 (26.29)	132	65.2	**0.20**	142	63.2	0.13
Generic module								
Total Score	154	59.2 (18.08)	132	67.2	**0.49**	141	67.7	**0.47**
Physical Summary	155	55.7 (23.98)	133	64.7	**0.43**	142	66.5	**0.42**
Psychosocial Summary	153	61.4 (18.07)	131	68.6	**0.43**	139	68.5	**0.40**
Emotional Functioning	153	60.5 (23.84)	131	70.2	**0.43**	140	71.4	**0.45**
Social Functioning	151	72.2 (22.00)	129	76.4	**0.24**	137	76.8	**0.27**
School Functioning	147	51.4 (22.55)	120	58.2	**0.33**	131	56.6	**0.25**

Effect sizes > or = 0.2 in bold are significant

The PedsQL™ Generic Core Scale and the PedsQL™ Multidimensional Fatigue Scale also had effect sizes representing a small to moderate response to change in all scales except for the cognitive fatigue domain for the one to three month follow up time point.

For the parent proxy measures, effect sizes were small to large. All scales of the proxy report of the PedsQL™ Sickle Cell Disease Module, Generic Core Scales, and Multidimensional Fatigue Scales were responsive to change except the Cognitive Fatigue scale (Additional file 1: Table S4).

#### Standard error of measurement

The SEM scores were stable and similar at one week and one to three months post discharge (Table 3). The SEM for the Total Scores on all three of the HRQL modules supports a change of 4–6 as a significant HRQL change. For the scales within each module, a change of 7–15 supports a relevant change in HRQL scores. Fifty-five to 66% of patients improved by one SEM or more in Total Scores in this study supporting the responsiveness of the measures for use during and after an acute painful crisis. Over 50% of patients improved in the three pain scales (Pain and Hurt, Pain Impact, and Pain Management) of the sickle cell disease specific module. The least improvement was in the Communication I domain (33% one week post discharge and 30% one to three months post discharge) of the disease specific module and in the School Functioning (40% one week post discharge and 36% one to three months post discharge) domain of the generic module.

**Table 3:** Percentage of patients with improved HRQL by 1 standard error of measurement or more

PedsQL™ HRQL Score	1 week post discharge			Steady state 1–3 months post-discharge		
Child self-report	N	SEM	Percentage with ≥1 SEM improvement	N	SEM	Percentage with ≥1 SEM improvement
Sickle cell disease module						
Total Score	139	3.94	65.5	153	3.80	66.0
Pain and Hurt	137	7.43	62.8	152	7.88	55.9
Pain Impact	139	7.00	64.0	151	7.50	58.9
Pain Management and Control	137	10.09	50.4	151	10.72	51.0
Worry I	137	9.74	47.4	151	9.68	60.3
Worry II	120	14.55	33.3	130	13.59	24.6
Emotions	138	12.89	40.6	152	14.03	42.8
Treatment	137	9.61	35.0	151	9.99	42.4
Communication I	136	12.27	33.1	149	11.49	30.2
Communication II	133	13.51	46.6	144	13.11	50.0
Fatigue module						
Total Score	134	5.72	54.5	144	5.49	54.2
General Fatigue	135	8.17	55.6	145	8.28	56.6
Sleep/Rest Fatigue	134	9.53	45.5	144	9.46	45.8
Cognitive Fatigue	132	8.20	47.0	142	8.04	45.1
Generic module						
Total Score	132	5.09	59.1	141	5.13	57.4
Physical Summary	133	8.07	53.4	142	8.03	54.2
Psychosocial Summary	131	6.28	55.0	139	6.39	51.8
Emotional Functioning	131	9.96	55.7	140	9.59	55.0
Social Functioning	129	9.62	42.6	137	9.33	44.5
School Functioning	120	10.22	40.0	131	11.09	35.9

For the parent proxy measures, a smaller change of 3–4.5 in the total HRQL scores supports a relevant HRQL change. For the scales within each module, a change of 5–12 supports a relevant change in HRQL scores.

### Anchor based method-Clinical change at one week post-discharge

#### PedsQL™ Sickle Cell Disease Module

The correlation coefficients between the global assessment of change in pain scores (anchor) and the HRQL Total scores (Sickle cell disease Total, Fatigue Total, and Generic Total. Pain and Hurt, Pain Impact, and Pain Management HRQL scores) ranged from 0.33–0.56 supporting the relevance of the anchor.

Examining change in HRQL scores for the pain scales (Pain and Hurt, Pain Impact, and Pain Management) with the anchor, the HRQL change score increased with each category of reported pain improvement (Table 4). Focusing on the change of “A little better” to represent the minimal patient reported improvement that supports a clinical change, the change in HRQL scores ranged from 7.5 to 10 for the three pain scales. This change is essentially the same change found using the SEM. A moderate to large improvement where a patient reported a change of “Much better” was reflected in a change in HRQL score of greater than 18 for the pain scales.

**Table 4:** Mean change in PedsQL™ health-related quality of life child report scores from baseline to one week post discharge as categorized by child’s perception of pain within the 7–10 days post discharge

Domain	No pain since discharge		Much better		A little better		The same or worse	
	N	Mean change (CI)^a^	N	Mean change (CI)^a^	N	Mean change (CI)^a^	N	Mean change (CI)^a^
PedsQL™ Sickle Cell Disease Module								
Total Score	19	20.1 (12.2, 28.0)	59	15.7 (10.6, 20.7)	41	5.8 (1.3, 10.3)	15	7.0 (0.4, 13.6)
Pain and Hurt	18	28.7 (17.8, 39.6)	59	25.1 (18.2, 32.1)	40	7.5 (1.1, 13.9)	15	3.9 (−7.0, 14.7)
Pain Impact	19	33.1 (21.3, 44.9)	58	19.8 (11.7, 28.0)	42	9.1 (3.5, 14.7)	15	8.7 (1.7, 15.7)
Pain Management and Control	18	18.8 (0.8, 36.7)	58	18.8 (10.9, 26.6)	42	9.8 (−0.3, 20.0)	14	6.3 (−8.7, 21.2)
PedsQL™ Multidimensional Fatigue Scale Total Score	18	10.8 (3.1, 18.5)	56	10.4 (5.2, 15.5)	41	4.8 (−0.8, 10.4)	14	0.1 (−10.0, 10.3)
PedsQL™ Generic Core Scales Total Score	18	9.5 (0.5, 18.6)	56	10.4 (5.4, 15.3)	39	9.5 (4.9, 14.2)	14	0.3 (−9.3, 9.9)

^a^95% Confidence interval

Examining the change in HRQL scores for the Total HRQL scores for each measure, a minimal improvement perceived by the patient as “a little better” is reflected as a change in HRQL score ranging from 4.8 (Fatigue Total score) to 9.5 (Generic Core Total score). These change scores are similar to the change score found when applying the SEM method.

For the parent proxy measures, higher change scores corresponded with improvement reported by the patient as “a little better” and “much better” for the sickle cell disease measure (Additional file 1: Table S6). For the fatigue and generic measures, the change in parent proxy HRQL scores were similar to the sickle cell disease measure for the different anchor categories.

#### Multivariable model: age, hydroxyurea use, site, gender and disease severity were not associated with improved HRQL over time

In the linear mixed-effects model, age, hydroxyurea use, site, gender, and disease severity were not significantly associated with change in HRQL (Table 5). HRQL did significantly improve from the time of presentation to the Emergency Department to one week post discharge even after adjustment for these covariates. Interactions between covariates and time were explored in other models, but were not found to be significant. Specifically, the change in HRQL over time is the same regardless of hydroxyurea use, age, hospital site, gender and disease severity.

**Table 5:** Multivariate analysis: Parameter estimates for child self-report of health-related quality of life over time

	One week post discharge	Age	Hydroxyurea
Total Score PedsQL™ Sickle Cell Disease Module	11.7 (<0.01)	−0.2 (0.31)	−1.2 (0.57)
Total Score PedsQL™ Multidimensional Fatigue Scale	7.7 (<0.01)	−0.3 (0.28)	−1.1 (0.65)
Total Score PedsQL™ Generic Core Scales	8.5 (<0.01)	−0.1 (0.60)	−2.6 (0.23)

(*p*-value)

#### Distribution curves

**Fig. 1:**
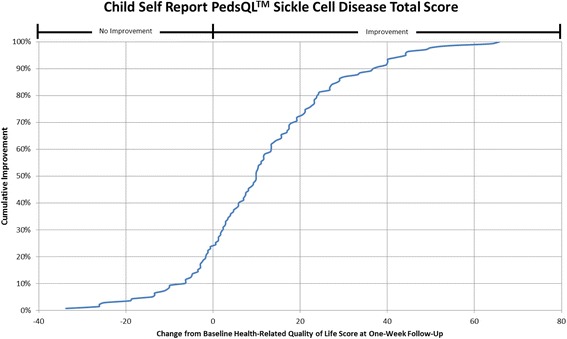
Cumulative Distribution Curve for the Child Self Report PedsQL™ Sickle Cell Disease Total Score

**Fig. 2:**
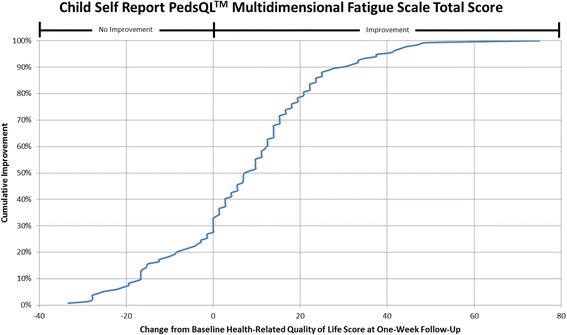
Cumulative Distribution Curve for the Child Self Report PedsQL™ Multidimensional Fatigue Scale Total Score

**Fig. 3:**
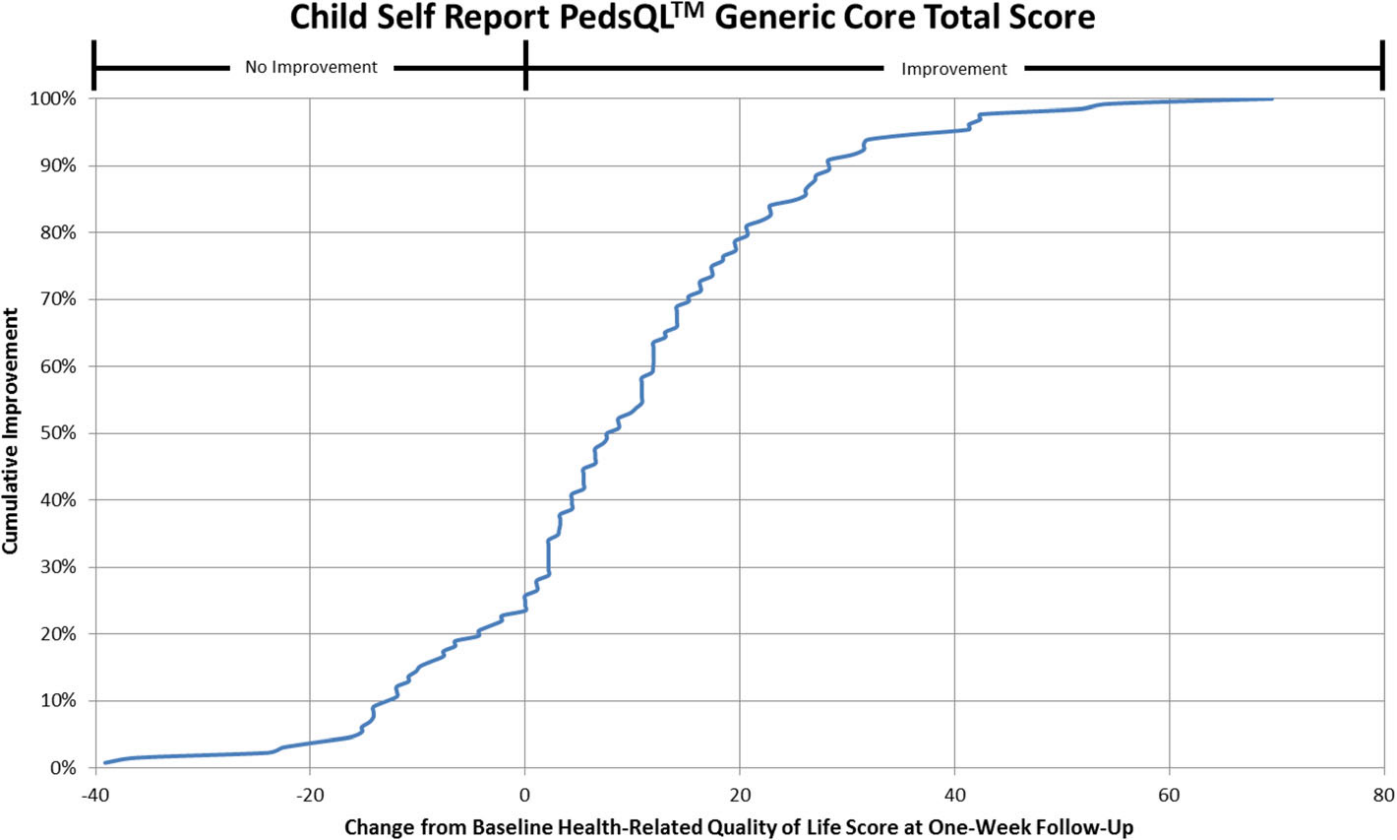
Cumulative Distribution Curve for the Child Self Report PedsQL™ Generic Core Total Score

**Fig. 4:**
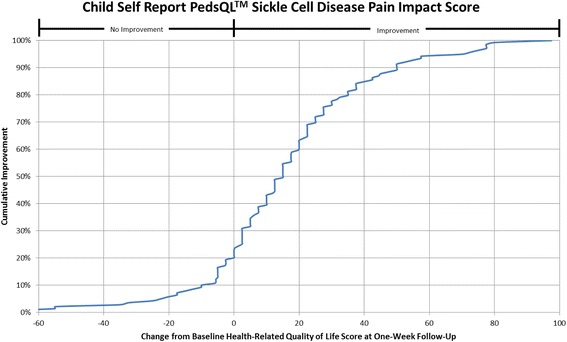
Cumulative Distribution Curve for the Child Self Report PedsQL™ Sickle Cell Disease Pain Impact Score

**Fig. 5:**
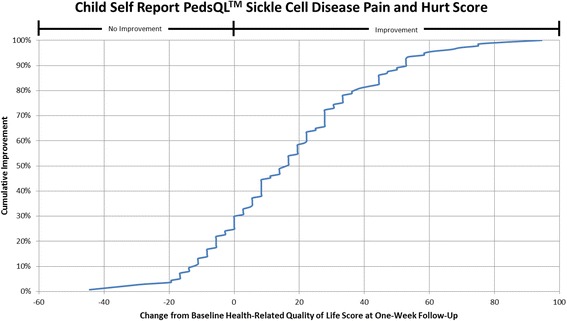
Cumulative Distribution Curve for the Child Self Report PedsQL™ Sickle Cell Disease Pain and Hurt Score

**Fig. 6:**
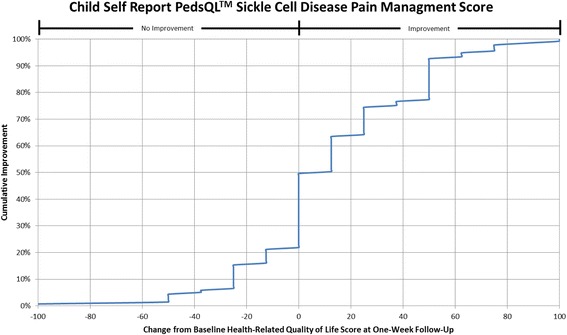
Cumulative Distribution Curve for the Child Self Report PedsQL™ Sickle Cell Disease Pain Management Score

The cumulative distribution curves which provide data needed to pick a responder threshold are shown for the Total HRQL score for each of the three measures in Figs. 1, 2 and 3 (Peds QL™ Sickle Cell Disease Module, the PedsQL™ Multidimensional Fatigue Scale and the PedsQL™ Generic Core Scales). In addition, Figs. 4, 5 and 6 show the cumulative distribution curves for the three pain scales of the Peds QL™ Sickle Cell Disease Module. Figure 1, for example, demonstrates that approximately 25% of patients had either no change in their total Sickle Cell Disease HRQL or had worse HRQL at one week post hospital discharge. In contrast, approximately 28% of patients had an improvement in total Sickle Cell Disease HRQL by 20 or more points at one week post discharge. See Additional file 2: Figures S1-S6 for parent proxy cumulative distribution curves.

## Discussion

The PedsQL™ Sickle Cell Disease Module is responsive to expected functional changes over time in patients with sickle cell disease experiencing acute painful vaso-occlusive crises. Our findings support the longitudinal validity of the PedsQL™ Sickle Cell Disease module. Specifically, the module is most responsive to change in the areas of pain, worry and communication. The PedsQL™ Multidimensional Fatigue module and PedsQL™ Generic Core scales were most responsive to change in general fatigue, sleep/rest, emotional functioning and physical functioning. Using both anchor and distribution based methods, our results also support relevant change scores for the module for patients experiencing acute painful vaso-occlusive crises. Lastly, our multivariable analyses confirm that patients with acute painful vaso-occlusive crises who are hospitalized have significant impairment in HRQL that improves in the week post discharge by which time it is similar to a patient’s baseline state at one to three months post discharge. This work provides the evidence needed to use patient reported outcomes in measuring treatment effectiveness in clinical trials and when estimating sample sizes for these trials. Given that patient reported outcomes are what patients usually care about most, understanding meaningful change scores is critical to patient centered care.

Our results indicate that the PedsQL™ Sickle Cell Disease module was responsive to changes over time for children hospitalized and treated for acute vaso-occlusive painful crises. This was true for all domains but Communication I domain. The Communication I domain asks about communicating with health care providers and wouldn’t be expected to change in this clinical setting over the period of time patients were followed. In addition, the PedsQL™ Multidimensional Fatigue scale was responsive to changes in all but the Cognitive Fatigue domain at one to three months post discharge. The Cognitive domain addresses issues with memory and attention whereby the General Fatigue and Sleep/Rest Fatigue domains focus on sleep and being physically active. Thus, it is understandable why significant changes over time were seen in the General Fatigue and Sleep/Rest Fatigue domains in this setting of recovery from an acute vaso-occlusive painful event. Lastly, the PedsQL™ Generic Core Scales were responsive to changes in all domains. These findings support the ability of these measures to detect change over time in patients with sickle cell disease who are experiencing acute vaso-occlusive crises.

Using a common and well accepted anchor approach, [15] we found that mean HRQL changes in specific HRQL domains that are less than 5–6 are likely not perceptible changes that reflect improvement in children recovering from acute painful vaso-occlusive crises. Furthermore, patients who perceived being “much better” had changes of 10–25 points in specific HRQL areas one week post hospital discharge. These meaningful changes in HRQL that we found using an anchor based approach are supported by the distribution methods we applied [16]. In addition, consistent with what has been described prior, the SEM in our study was similar across time points as it accounts for the variability of the data [8, 9, 17].

Cumulative distribution curves allow one to see the full spectrum of HRQL change and to determine the threshold of change that is appropriate for future research and in clinical care. This prevents one from having to rely on what can be a more arbitrary change threshold. This is important when studying pain given the inherent variability in pain expression from person to person. In our study, approximately 40% of patients had a change score in Total Sickle Cell Disease HRQL of 5.8 (perceived by patients as a little better) or less at one week post discharge. Sixty-two percent of patients had a change score in Total Sickle Cell Disease HRQL of 15.7 (perceived by patients as much better) or less. Thus, the value of reporting the cumulative distribution curves allows for comparison of HRQL change across thresholds. In addition, this facilitates comparison across thresholds to determine treatment impact. For example, the percentage of patients who have a change of at least the minimum can be compared across different treatment groups to determine the effectiveness of therapy on patient reported outcomes.

It is important to remember that meaningful change in HRQL varies by population and context. Our study is limited to patients with sickle cell disease who were hospitalized with acute painful vaso-occlusive crises and may not be generalizable to all patients such as to those who experience chronic pain. Further work is needed to determine if the PedsQL™ Sickle Cell Disease module is responsive to changes in patient reported well being in other settings. For example, it is unknown if the measure would detect changes in the well being of patients treating painful events at home. In addition, this study used a global change in pain question as the anchor for meaningful change in HRQL. Although this is the most common method used and is a valid anchor to use, there is the risk of recall bias or response shift as patients are asked to retrospectively report their change in health [10]. Additionally, this anchor is not relevant to a patient’s changes in other HRQL domains such as the emotion domain and further evidence is needed to determine the meaningful change for these domains. Lastly, there was a small subset of patients who reported their pain was no better or worse than when they were initially seen in the Emergency Department. Despite this, as a group, these patients had improvement in their Total Sickle Cell Disease HRQL score reflecting most likely that the patients are improved in other areas of functioning. When examining the pain domains, the change in HRQL reflecting patients who reported they were “a little better” one week post discharge for both the Pain and Hurt and Pain Management domains was 7.5 and 9.8 respectively. In comparison, the change in HRQL reflecting patients that were “the same or worse” was 3.9 for the Pain and Hurt domain and 6.3 for the Pain Management domain supporting a relevant change in HRQL for these two domains.

## Conclusions

In summary, our study provides the longitudinal validity and meaningful change in HRQL scores for relevant domains of the PedsQL Sickle Cell Disease module, the PedsQL™ Multidimensional Fatigue scale, and PedsQL™ Generic Core Scales. These results are applicable to understanding the clinical use of HRQL measures to aid in evaluating patients’ functioning over time and may also inform future clinical trial research to measure effectiveness of interventions in children with sickle cell disease.

## Additional files

Additional file 1: Table S1. CONSORT PRO PedsQL™ Sickle Cell Disease Module Completion Data. **Table S2.** CONSORT PRO Outcome Data for PedsQL™ Multi-dimensional Fatigue Module Completion Data. **Table S3.** CONSORT PRO Outcome Data for PedsQL™ Generic Core Scales Completion Data. **Table S4.** Parent Proxy Report Effect Size. **Table S5.** Percentage of patients by parent proxy report with improved HRQL by 1 standard error of measurement or more. **Table S6.** Mean change in PedsQL™ health-related quality of life parent proxy scores from baseline to 7–10 days post discharge as categorized by child’s perception of pain within the 7–10 days post discharge. (DOCX 41 kb)

Additional file 2: Figure S1. Cumulative Distribution Curve for the Parent Proxy Report PedsQL™ Sickle Cell Disease Total Score. **Figure S2.** Cumulative Distribution Curve for the Parent Proxy Report PedsQL™ Multidimensional Fatigue Scale Total Score. **Figure S3.** Cumulative Distribution Curve for the Parent Proxy Report PedsQL™ Generic Core Total Score. **Figure S4.** Cumulative Distribution Curve for the Parent Proxy Report PedsQL™ Sickle Cell Disease Pain Impact Score. **Figure S5.** Cumulative Distribution Curve for the Parent Proxy Report PedsQL™ Sickle Cell Disease Pain and Hurt Score. **Figure S6.** Cumulative Distribution Curve for the Parent Proxy Report PedsQL™ Sickle Cell Disease Pain Management Score. (DOCX 328 kb)

## Abbreviations

HRQL: Health-related quality of life
SEM: Standard error of measurement
