# Supplementary material for: Determining the longitudinal validity and meaningful differences in HRQL of the PedsQL™ Sickle Cell Disease Module
Source: Health Qual Life Outcomes. 2017 Jun 12;15:124. doi: 10.1186/s12955-017-0700-2 (PMC5468970; doi:10.1186/s12955-017-0700-2)
Supplement: Supplementary file 1 — CONSORT PRO PedsQL™ Sickle Cell Disease Module Completion Data. Table S2. CONSORT PRO Outcome Data for PedsQL™ Multi-dimensional Fatigue Module Completion Data. Table S3. CONSORT PRO Outcome Data for PedsQL™ Generic Core Scales Completion Data. Table S4. Parent Proxy Report Effect Size. Table S5. Percentage of patients by parent proxy report with improved HRQL by 1 standard error of measurement or more. Table S6. Mean change in PedsQL™ health-related quality of life parent proxy scores from baseline to 7–10 days post discharge as categorized by child’s perception of pain within the 7–10 days post discharge. (DOCX 41 kb) [file 12955_2017_700_MOESM1_ESM.docx]

**Table S1.** CONSORT PRO PedsQL™ Sickle Cell Disease Module

Completion Data

|  | **Baseline: Emergency Department** | **One week follow-up** | **One-to-three month follow-up** |
| --- | --- | --- | --- |
| **Child self-report PedsQL™**  **Sickle Cell Disease Module** |  |  |  |
| Total enrolled at study time point | 187 | 187 | 187 |
| Completed | 166 | 154 | 170 |
| Missing | 21 | 33 | 17 |
| Partially completed  (unscoreable) | 1 | 1 | 1 |
| Lost to follow-up at study  time point | 0 | 20 | 12 |
| Did not complete survey | 20 | 12 | 4 |
|  |  |  |  |
| **Parent proxy report PedsQL™ Sickle Cell Disease Module** |  |  |  |
| Total enrolled at study time point | 187 | 187 | 187 |
| Completed | 147 | 139 | 149 |
| Missing | 40 | 48 | 38 |
| Partially completed  (unscoreable) | 0 | 2 | 0 |
| Lost to follow-up at study  time point | 0 | 11 | 12 |
| Did not complete survey | 40 | 35 | 26 |

**Table S2.** CONSORT PRO Outcome Data for PedsQL™ Multi-dimensional Fatigue Module Completion Data

|  | **Baseline: Emergency Department** | **One week follow-up** | **One-to-three month follow-up** |
| --- | --- | --- | --- |
| **Child self-report PedsQL™**  **Multi-dimensional Fatigue Scales** |  |  |  |
| Total enrolled at study time point | 187 | 187 | 187 |
| Completed | 157 | 154 | 170 |
| Missing | 30 | 33 | 17 |
| Partially completed  (unscoreable) | 10 | 1 | 1 |
| Lost to follow-up at study  time point | 0 | 20 | 12 |
| Did not complete survey | 20 | 12 | 4 |
|  |  |  |  |
| **Parent proxy report PedsQL™ Multi-dimensional Fatigue Scales** |  |  |  |
| Total enrolled at study time point | 187 | 187 | 187 |
| Completed | 147 | 140 | 148 |
| Missing | 40 | 47 | 39 |
| Partially completed  (unscoreable) | 0 | 1 | 1 |
| Lost to follow-up at study  time point | 0 | 11 | 12 |
| Did not complete survey | 40 | 35 | 26 |

**Table S3.** CONSORT PRO Outcome Data for PedsQL™ Generic Core

Scales Completion Data

|  | **Baseline: Emergency Department** | **One week follow-up** | **One-to-three month follow-up** |
| --- | --- | --- | --- |
| **Child self-report PedsQL™**  **Generic Core Scales** |  |  |  |
| Total enrolled at study time point | 187 | 187 | 187 |
| Completed | 154 | 155 | 170 |
| Missing | 33 | 32 | 17 |
| Partially completed  (unscoreable) | 13 | 0 | 1 |
| Lost to follow-up at study  time point | 0 | 20 | 12 |
| Did not complete survey | 20 | 12 | 4 |
|  |  |  |  |
| **Parent proxy report PedsQL™ Generic Core Scales** |  |  |  |
| Total enrolled at study time point | 187 | 187 | 187 |
| Completed | 146 | 139 | 149 |
| Missing | 41 | 48 | 38 |
| Partially completed  (unscoreable) | 1 | 2 | 0 |
| Lost to follow-up at study  time point | 0 | 11 | 12 |
| Did not complete survey | 40 | 35 | 26 |

**Table S4.** Parent Proxy Report Effect Size

|  | **ED Visit** | | **1 Week** | | | | **1-3 Months** | | | |
| --- | --- | --- | --- | --- | --- | --- | --- | --- | --- | --- |
| **Domain** | **N** | **Mean (SD)** | **N** | **Mean** | | **Effect Size** | **N** | **Mean** | | **Effect Size** |
| **Sickle cell disease**  **module** |  |  |  | |  |  |  | |  |  |
| Total Score | 147 | 51.9 (17.14) | 131 | | 70.0 | **1.04** | 139 | | 65.3 | **0.80** |
| Pain and Hurt | 147 | 52.2 (19.58) | 132 | | 73.7 | **1.13** | 139 | | 65.9 | **0.73** |
| Pain Impact | 147 | 36.1 (21.03) | 131 | | 59.8 | **1.20** | 139 | | 56.5 | **1.01** |
| Pain Management  and Control | 147 | 42.3 (24.36) | 132 | | 62.3 | **0.87** | 137 | | 59.6 | **0.74** |
| Worry I | 147 | 55.6 (27.19) | 131 | | 70.3 | **0.56** | 139 | | 68.7 | **0.46** |
| Worry II | 146 | 70.7 (30.84) | 130 | | 82.9 | **0.43** | 138 | | 80.2 | **0.32** |
| Emotions | 147 | 49.5 (31.61) | 132 | | 62.2 | **0.41** | 137 | | 61.4 | **0.36** |
| Treatment | 147 | 56.5 (22.35) | 132 | | 67.4 | **0.51** | 137 | | 65.3 | **0.37** |
| Communication I | 147 | 76.2 (23.33) | 132 | | 85.1 | **0.37** | 137 | | 81.1 | **0.22** |
| Communication II | 146 | 57.7 (27.26) | 131 | | 69.4 | **0.42** | 136 | | 67.7 | **0.38** |
| **Fatigue module** |  |  |  | |  |  |  | |  |  |
| Total Score | 147 | 58.4 (19.60) | 132 | | 72.1 | **0.76** | 138 | | 66.9 | **0.42** |
| General Fatigue | 146 | 53.0 (22.70) | 131 | | 70.5 | **0.82** | 138 | | 65.7 | **0.54** |
| Sleep/Rest Fatigue | 147 | 54.8 (23.03) | 131 | | 71.3 | **0.75** | 138 | | 66.8 | **0.52** |
| Cognitive Fatigue | 147 | 67.1 (25.85) | 132 | | 74.8 | **0.34** | 138 | | 68.3 | 0.03 |
| **Generic module** |  |  |  | |  |  |  | |  |  |
| Total Score | 146 | 61.7 (18.44) | 130 | | 75.9 | **0.82** | 138 | | 70.8 | **0.49** |
| Physical Summary | 146 | 57.7 (22.07) | 129 | | 74.4 | **0.80** | 138 | | 68.7 | **0.50** |
| Psychosocial  Summary | 145 | 64.0 (19.25) | 129 | | 76.7 | **0.71** | 137 | | 71.9 | **0.40** |
| Emotional  Functioning | 145 | 64.3 (22.29) | 129 | | 78.2 | **0.64** | 131 | | 75.8 | **0.48** |
| Social Functioning | 146 | 72.8 (23.49) | 130 | | 82.9 | **0.47** | 138 | | 78.4 | **0.25** |
| School Functioning | 141 | 54.1 (23.32) | 120 | | 68.1 | **0.59** | 131 | | 61.1 | **0.30** |

**Table S5.** Percentage of patients by parent proxy report with improved HRQL by 1 standard error of measurement or more

| **PedsQL™HRQL Score** | **1 week post-discharge** | | | **1-3 months post-discharge** | | |
| --- | --- | --- | --- | --- | --- | --- |
| **Parent proxy-report** | **SEM** | **Percentage with ≥ SEM** | **N**  **1 week** | **SEM** | **Percentage with ≥ SEM improvement** | **N**  **1-3 month** |
| **Sickle cell disease module** |  |  |  |  |  |  |
| Total SCD | 3.61 | 78.6 | 131 | 3.28 | 70.5 | 139 |
| Pain and hurt | 6.69 | 78.8 | 132 | 5.85 | 62.6 | 139 |
| Pain impact | 6.18 | 71.0 | 131 | 5.20 | 71.2 | 139 |
| Pain management | 7.83 | 60.6 | 132 | 5.71 | 56.2 | 137 |
| Worry I | 8.03 | 63.4 | 131 | 7.07 | 53.2 | 139 |
| Worry II | 10.67 | 46.9 | 130 | 9.79 | 41.3 | 138 |
| Emotions | 12.38 | 56.1 | 132 | 12.33 | 50.4 | 137 |
| Treatment | 8.68 | 50.8 | 132 | 7.90 | 40.1 | 137 |
| Communication I | 7.03 | 43.2 | 132 | 7.16 | 38.7 | 137 |
| Communication II | 10.29 | 43.5 | 131 | 10.11 | 40.4 | 136 |
| **Fatigue module** |  |  |  |  |  |  |
| Total Fatigue | 4.52 | 68.2 | 132 | 4.31 | 52.9 | 138 |
| General fatigue | 6.51 | 66.4 | 131 | 6.04 | 55.1 | 138 |
| Sleep/Rest fatigue | 7.12 | 63.4 | 131 | 6.64 | 57.2 | 138 |
| Cognitive fatigue | 5.30 | 49.2 | 132 | 4.88 | 32.6 | 138 |
| **Generic module** |  |  |  |  |  |  |
| Total Generic | 4.24 | 70.0 | 130 | 4.26 | 58.7 | 138 |
| Physical summary | 6.85 | 64.6 | 130 | 6.91 | 51.4 | 138 |
| Psychosocial summary | 5.08 | 62.0 | 129 | 5.06 | 49.6 | 137 |
| Emotional Functioning | 7.75 | 60.5 | 129 | 7.58 | 50.4 | 137 |
| Social Functioning | 8.18 | 49.2 | 130 | 8.37 | 42.8 | 138 |
| School Functioning | 7.55 | 60.0 | 120 | 7.00 | 42.0 | 131 |

**Table S6.** Mean change in PedsQL™ health-related quality of life parent proxy scores from baseline to 7-10 days post discharge as categorized by child’s perception of pain within the 7-10 days post discharge

|  | **No pain since discharge** | | | **Much better** | | **A little better** | | **The same or worse** | | |
| --- | --- | --- | --- | --- | --- | --- | --- | --- | --- | --- |
|  | **N** | **Mean change (CI)*** | | **N** | **Mean change (CI)*** | **N** | **Mean change (CI)*** | **N** | | **Mean change (CI)*** |
| **PedsQL™ Sickle Cell Disease**  **Module** | | | | | | | | | | |
| Total Score | 16 | 26.7 (20.2, 33.3) | 54 | | 20.1 (14.3, 25.8) | 32 | 15.2 (9.8, 20.5) | | 13 | 5.3 (-5.1, 15.6) |
| Pain and hurt | 16 | 28.6 (22.7, 34.4) | 54 | | 24.8 (18.0, 31.6) | 32 | 22.9 (14.6, 31.2) | | 13 | 5.3 (-5.6, 16.3) |
| Pain impact | 16 | 42.5 (29.4, 55.6) | 54 | | 28.9 (20.0, 37.9) | 32 | 15.9 (8.4, 23.4) | | 13 | 11.0 (-7.8, 29.7) |
| Pain management | 17 | 34.6 (21.4, 47.7) | 54 | | 23.8 (14.2, 33.5) | 32 | 19.9 (9.2, 30.7) | | 13 | 3.8 (-18.7, 26.4) |
|  | | | | | | | | | | |
| **PedsQL™ Multidimensional Fatigue Scale Total Score** | 17 | 18.0 (8.5, 27.5) | 54 | | 16.6 (11.0, 22.2) | 32 | 17.2 (9.4, 25.0) | | 13 | 6.6 (-4.9, 18.0) |
|  |  |  |  | |  |  |  | |  |  |
| **PedsQL™ Generic Core Scales Total Score** | 17 | 20.3 (11.8, 28.7) | 52 | | 16.8 (11.5, 22.0) | 32 | 17.8 (10.7, 24.9) | | 13 | 1.2 (-9.4, 11.7) |
|  |  |  |  | |  |  |  | |  |  |
| *95% Confidence interval | | | | | | | | | | |
