# Supplementary material for: Determining the longitudinal validity and meaningful differences in HRQL of the PedsQL™ Sickle Cell Disease Module
Source: Health Qual Life Outcomes. 2017 Jun 12;15:124. doi: 10.1186/s12955-017-0700-2 (PMC5468970; doi:10.1186/s12955-017-0700-2)

Figure S1. Cumulative Distribution Curve for the Parent Report PedsQL™ Sickle Cell Disease Total Score


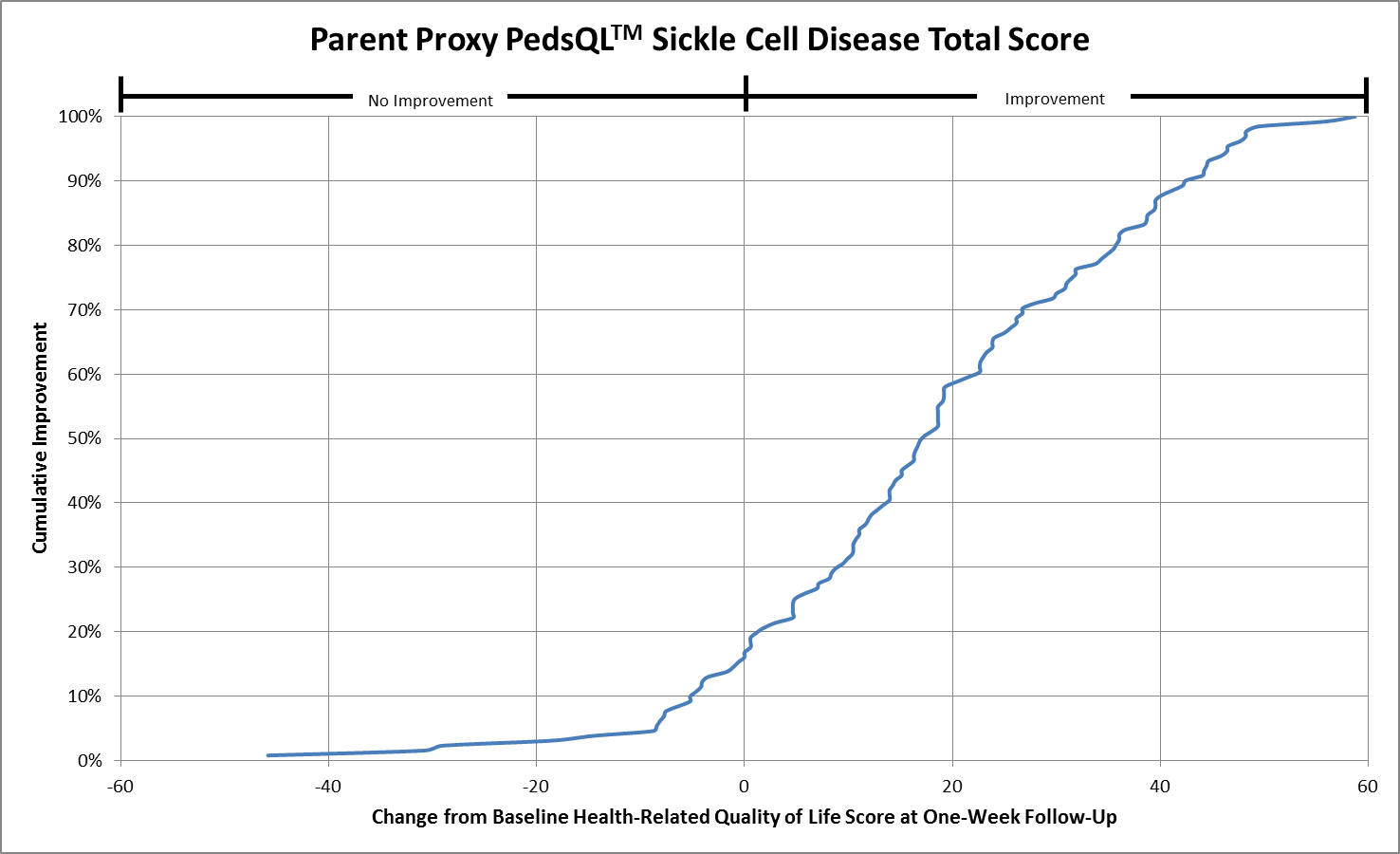


Figure S2. Cumulative Distribution Curve for the Parent Report PedsQL™ Multidimensional Fatigue Scale Total Score


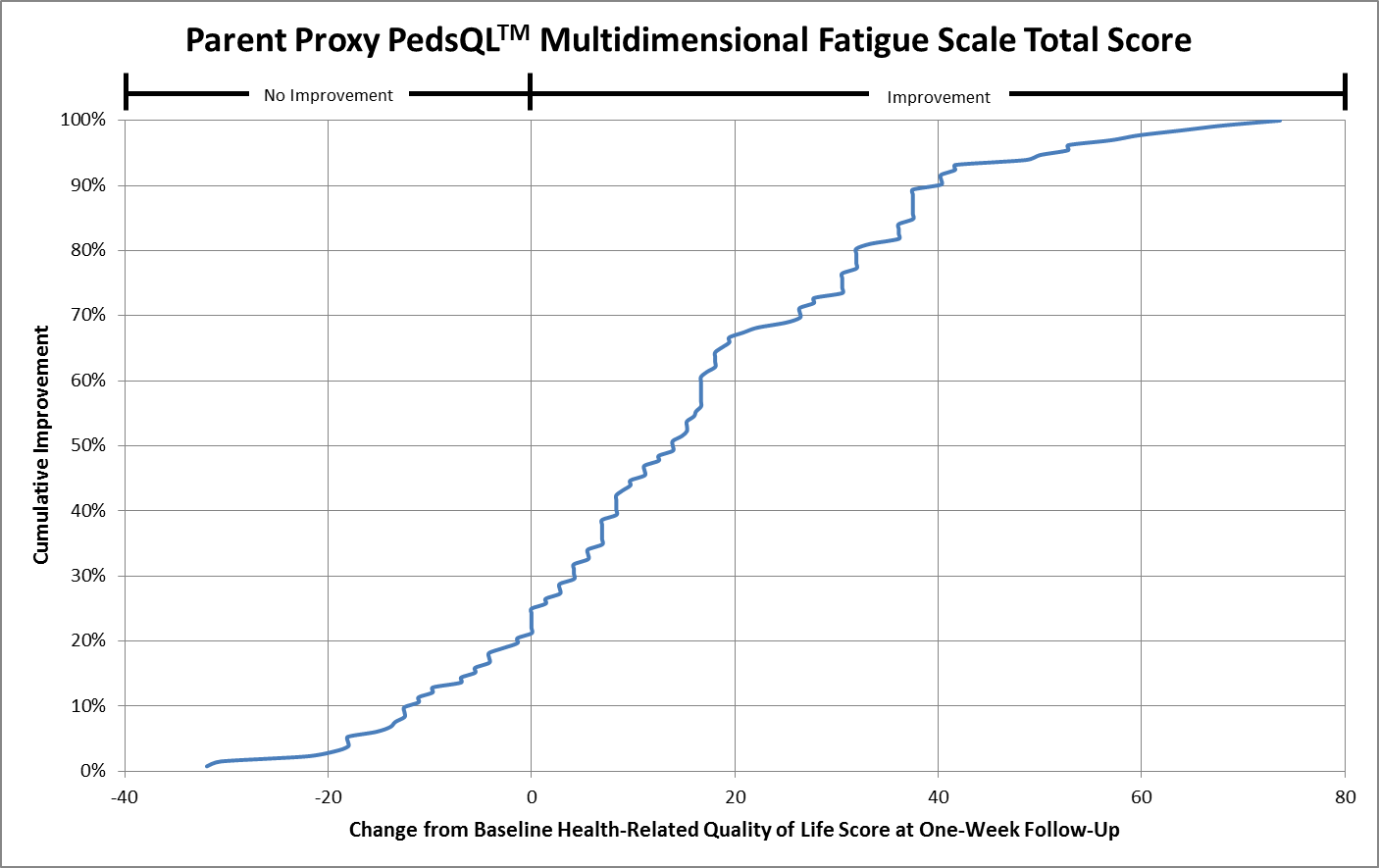


Figure S3. Cumulative Distribution Curve for the Parent Report PedsQL™ Generic Core Total Score


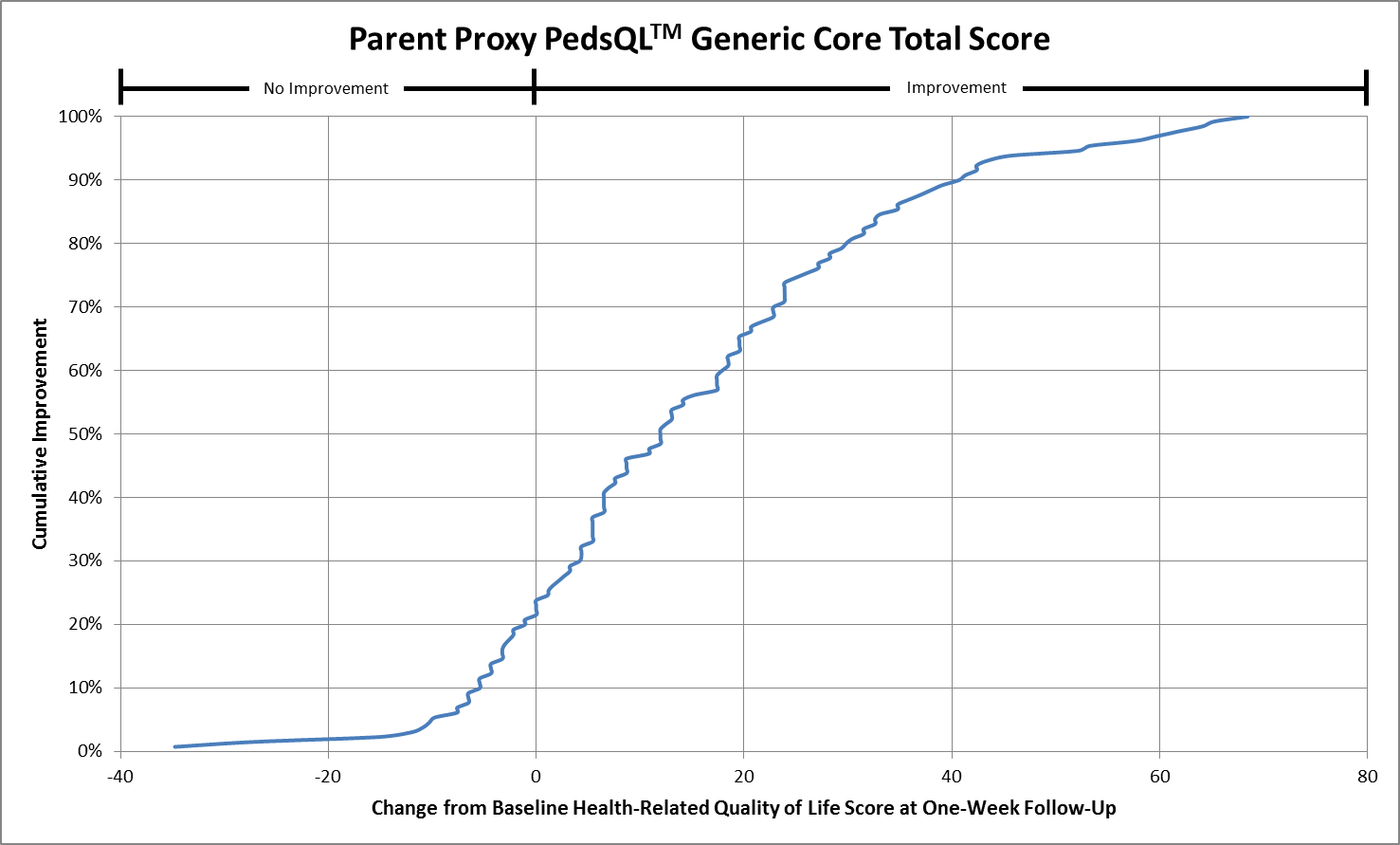


Figure S4. Cumulative Distribution Curve for the Parent Report PedsQL™ Sickle Cell Disease Pain Impact Score


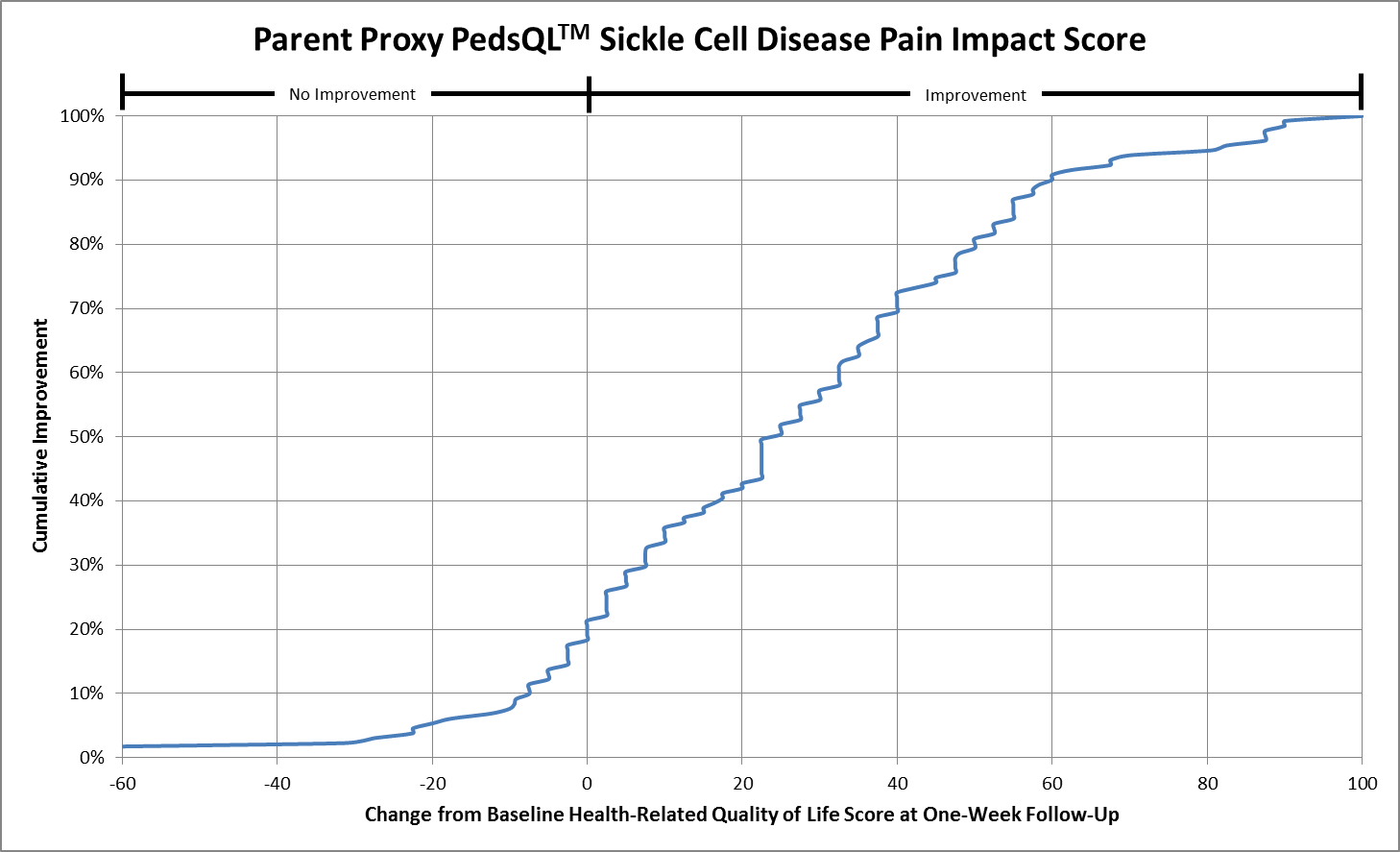


Figure S5. Cumulative Distribution Curve for the Parent Report PedsQL™ Sickle Cell Disease Pain and Hurt Score


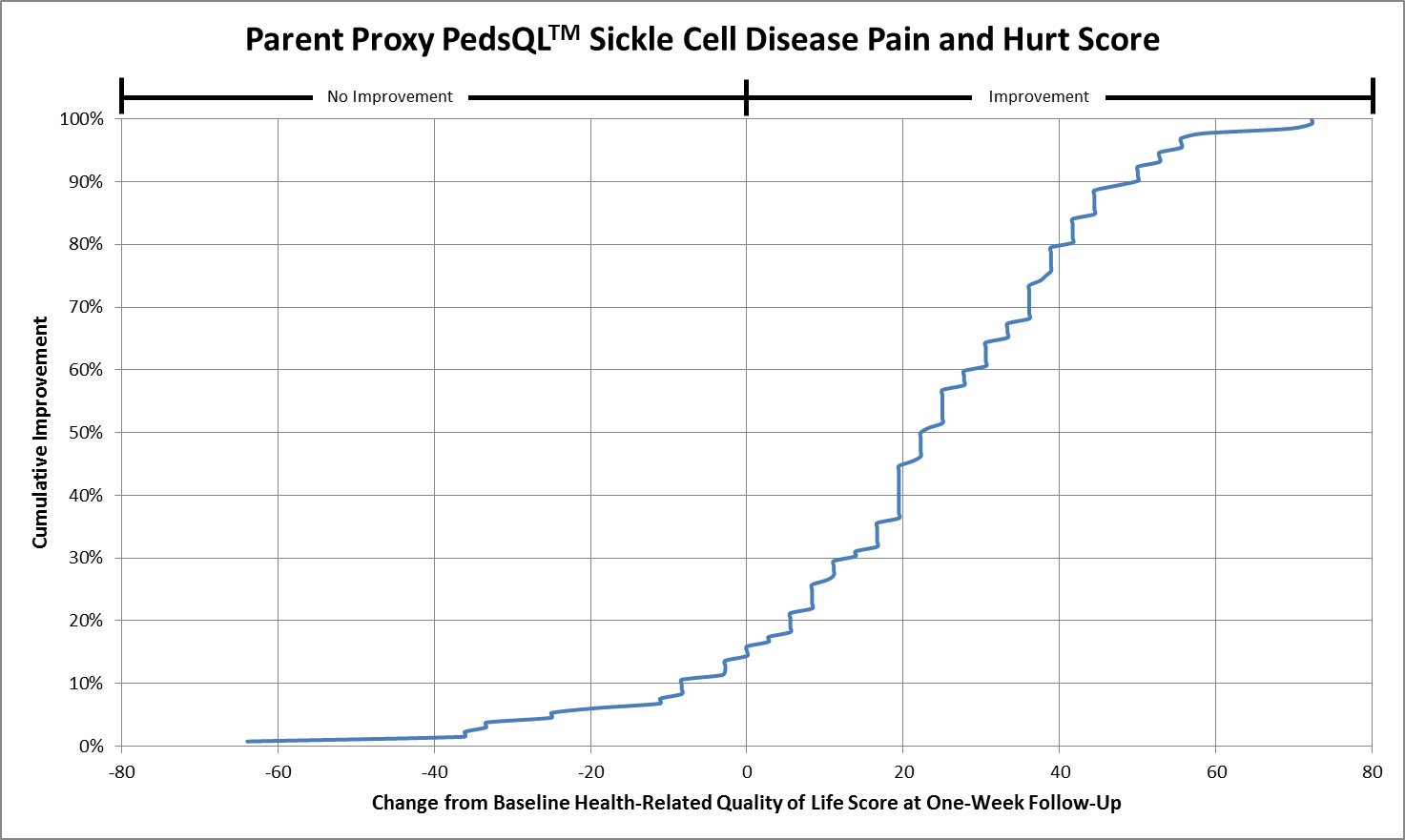


Figure S6. Cumulative Distribution Curve for the Parent Report PedsQL™ Sickle Cell Disease Pain Management Score


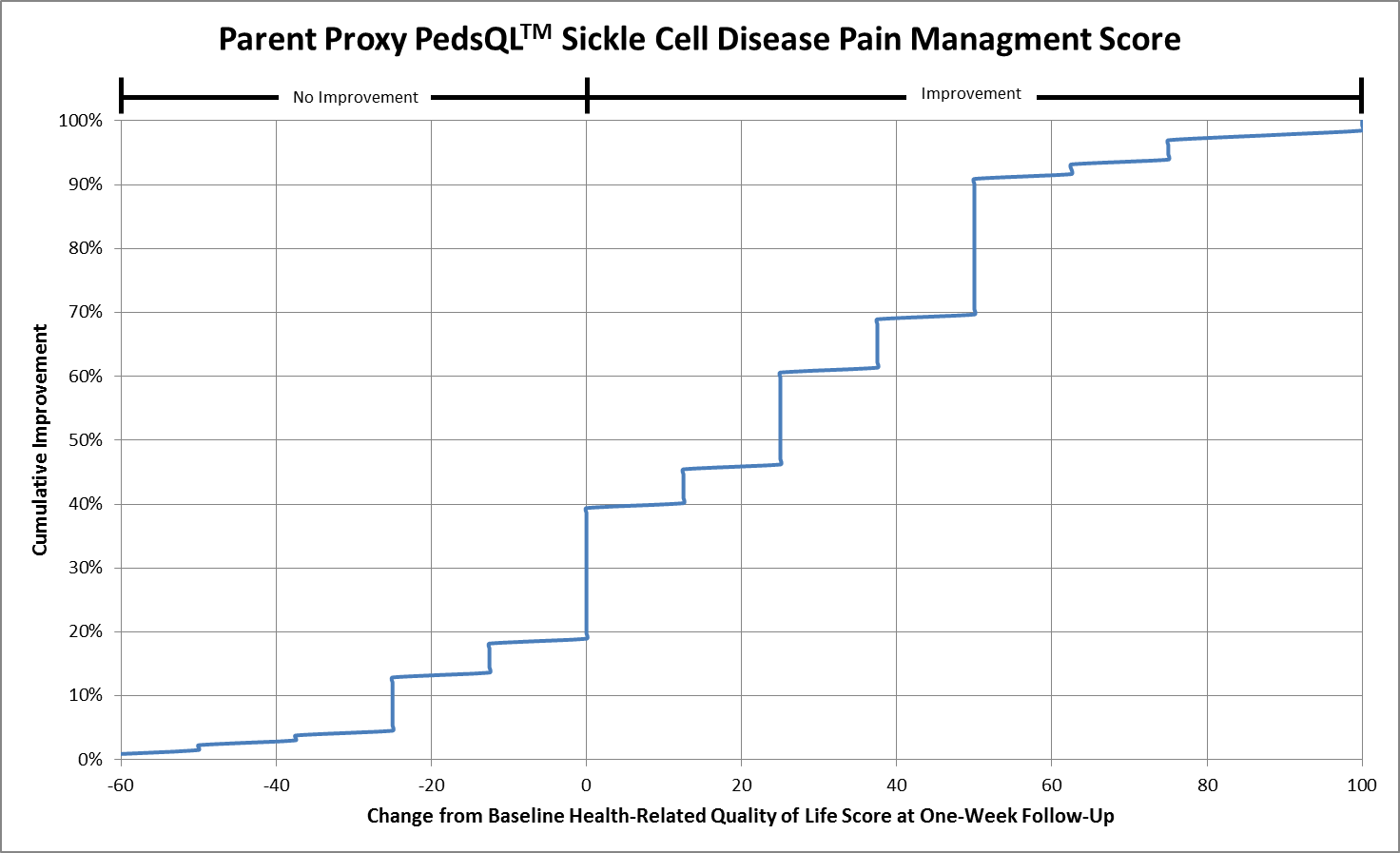

Supplement: Supplementary file 2 — Cumulative Distribution Curve for the Parent Proxy Report PedsQL™ Sickle Cell Disease Total Score. Figure S2. Cumulative Distribution Curve for the Parent Proxy Report PedsQL™ Multidimensional Fatigue Scale Total Score. Figure S3. Cumulative Distribution Curve for the Parent Proxy Report PedsQL™ Generic Core Total Score. Figure S4. Cumulative Distribution Curve for the Parent Proxy Report PedsQL™ Sickle Cell Disease Pain Impact Score. Figure S5. Cumulative Distribution Curve for the Parent Proxy Report PedsQL™ Sickle Cell Disease Pain and Hurt Score. Figure S6. Cumulative Distribution Curve for the Parent Proxy Report PedsQL™ Sickle Cell Disease Pain Management Score. (DOCX 328 kb) [file 12955_2017_700_MOESM2_ESM.docx]
